# (μ-3-Acetyl-5-carboxyl­ato-4-methyl­pyrazolido-1:2κ^4^ *N* ^2^,*O* ^3^:*N* ^1^,*O* ^5^)-μ-chlorido-tetra­pyridine-1κ^2^ *N*,2κ^2^ *N*-chlorido-1κ*Cl*-dicopper(II) propan-2-ol solvate

**DOI:** 10.1107/S1600536809038276

**Published:** 2009-09-26

**Authors:** Sergey Malinkin, Larisa Penkova, Vadim A. Pavlenko, Matti Haukka, Igor O. Fritsky

**Affiliations:** a Department of Chemistry, Kiev National Taras Shevchenko University, Volodymyrska Street 64, 01601 Kiev, Ukraine; b Department of Chemistry, University of Joensuu, PO Box 111, FI-80101 Joensuu, Finland

## Abstract

The title compound, [Cu_2_(C_7_H_6_N_2_O_3_)Cl_2_(C_5_H_5_N)_4_]·C_3_H_8_O, is a binuclear pyrazolate complex, in which the two Cu^II^ atoms have different coordination numbers and are connected by a bridging Cl atom. One Cu^II^ atom has a distorted square-pyramidal coordination environment formed by two pyridine N atoms, one bridging Cl atom and an *N*,*O*-chelating pyrazolate ligand. The other Cu^II^ atom adopts an octa­hedral geometry defined by two pyridine N atoms at the axial positions, two Cl atoms and the coordinated pyrazolate ligand in the equatorial plane. An O—H⋯O hydrogen bond connects the complex mol­ecules and propan-2-ol solvent mol­ecules into pairs. These pairs form columns along the *a* axis.

## Related literature

For other 3,5-substituted-1*H*-pyrazolate complexes, see: Driessen *et al.* (2003 ▶); Eisenwiener *et al.* (2007 ▶); King *et al.* (2004 ▶); Li (2005 ▶); Penkova *et al.* (2008 ▶); Tretyakov *et al.* (2008 ▶).
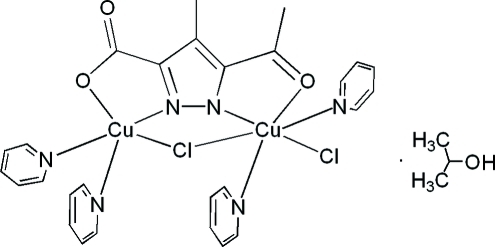


## Experimental

### 

#### Crystal data

[Cu_2_(C_7_H_6_N_2_O_3_)Cl_2_(C_5_H_5_N)_4_]·C_3_H_8_O
*M* *_r_* = 740.61
Monoclinic, 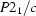

*a* = 16.4130 (4) Å
*b* = 12.6351 (2) Å
*c* = 16.5739 (4) Å
β = 107.2145 (12)°
*V* = 3283.12 (12) Å^3^
*Z* = 4
Mo *K*α radiation
μ = 1.50 mm^−1^
*T* = 100 K
0.24 × 0.16 × 0.13 mm

#### Data collection

Nonius KappaCCD diffractometer
Absorption correction: multi-scan (*SADABS*; Sheldrick, 1996 ▶) *T*_min_ = 0.718, *T*_max_ = 0.832
45344 measured reflections
8719 independent reflections
6355 reflections with *I* > 2σ(*I*)
*R*_int_ = 0.058

#### Refinement

*R*[*F* ^2^ > 2σ(*F* ^2^)] = 0.038
*wR*(*F* ^2^) = 0.086
*S* = 1.03
8719 reflections
402 parameters
H-atom parameters constrained
Δρ_max_ = 0.59 e Å^−3^
Δρ_min_ = −0.47 e Å^−3^

### 

Data collection: *COLLECT* (Nonius, 1998 ▶); cell refinement: *DENZO*/*SCALEPACK* (Otwinowski & Minor, 1997 ▶); data reduction: *DENZO*/*SCALEPACK*; program(s) used to solve structure: *SIR2004* (Burla *et al.*, 2005 ▶); program(s) used to refine structure: *SHELXL97* (Sheldrick, 2008 ▶); molecular graphics: *ORTEP-3* (Farrugia, 1997 ▶); software used to prepare material for publication: *SHELXL97*.

## Supplementary Material

Crystal structure: contains datablocks I, global. DOI: 10.1107/S1600536809038276/hy2229sup1.cif

Structure factors: contains datablocks I. DOI: 10.1107/S1600536809038276/hy2229Isup2.hkl

Additional supplementary materials:  crystallographic information; 3D view; checkCIF report

## Figures and Tables

**Table 1:** Selected bond lengths (Å)

Cu1—N1	1.9814 (18)
Cu1—N3	2.0609 (18)
Cu1—N4	2.0371 (18)
Cu1—O1	2.5878 (17)
Cu1—Cl1	2.2634 (6)
Cu1—Cl2	2.8621 (6)
Cu2—N2	1.9549 (18)
Cu2—N5	2.0097 (18)
Cu2—N6	2.1987 (18)
Cu2—O2	2.0340 (16)
Cu2—Cl2	2.3036 (6)

**Table 2:** Hydrogen-bond geometry (Å, °)

*D*—H⋯*A*	*D*—H	H⋯*A*	*D*⋯*A*	*D*—H⋯*A*
O4—H3*O*⋯O3	0.95	1.82	2.734 (3)	160

**Fig. 1.**
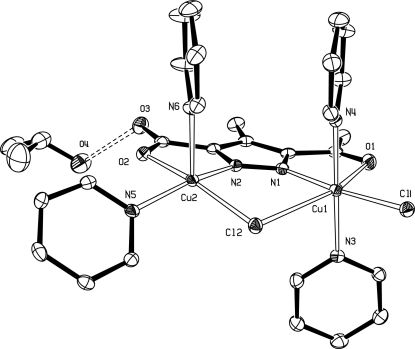
A view of the title compound. Displacement ellipsoids are shown at the 50% probability level. Hydrogen bonds are indicated by dashed lines. H atoms have been omitted for clarity.

**Fig. 2.**
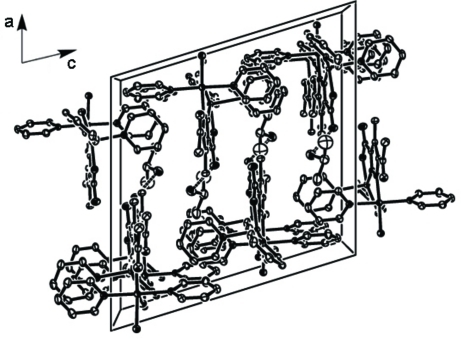
Crystal packing of the title compound.

**Fig. 3.**
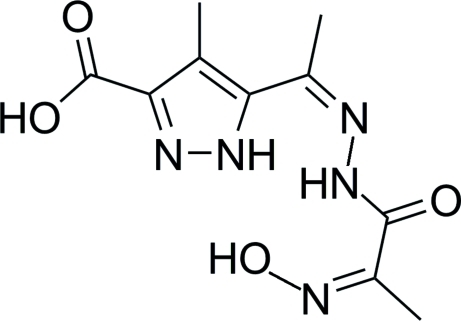
The structural formula of L.

## supplementary crystallographic information

### Comment

Pyrazole-based chelating ligands form a variety of coordination complexes providing various coordination geometries and nuclearities (Eisenwiener *et al.*, 2007). In the synthesis of supramolecular inorganic architectures by design, the assembly of molecular units in predefined arrangements is a key goal (Tretyakov *et al.*, 2008). Linear bi- and trinuclear copper(II) complexes are of interest as models for the active sites of multicopper proteins, like ascorbate oxidase, ceruloplasmin and laccase (Driessen *et al.*, 2003), and are also of interest for a better understanding of the magnetic properties of multicopper compounds (Penkova *et al.*, 2008). The preparation and crystal structure of the title compound, a novel binuclear pyrazolate complex based on 5-acetyl-4-methyl-1H-pyrazol-3-carboxylic acid incorporating two Cu centres in different coordination environments, are reported herein. The complex was obtained as a product of the hydrolytic cleavage of L (see Scheme 2) in the presence of Cu ions.

In the molecular structure, the Cu^II^ atoms adopt different types of coordination geometries (Fig. 1). The geometry around Cu2 is distorted square-pyramidal. The Cu2—N2 bond distance is 1.9549 (18) Å (Table 1), close to those observed in the pyrazolato-bridged, linear trinuclear Cu^II^ complex reported by Driessen *et al.* (2003) [average Cu—N = 1.965 (5) Å]. The carboxylate group is in the basal plane with Cu2—O2 distance similar to that observed in the structure reported by Li (2005) [Cu—O = 2.016 (3) Å]. The N atom of a pyridine molecule occupies the apical position with Cu2—N6 distance of 2.1987 (18) Å. The Cu1 atom is situated in a slightly distorted octahedral environment formed by two N atoms belonging to the pyridine molecules occupying the axial positions, two Cl atoms (one of which is bridging) and N and O atoms of the pyrazolate ligand providing an N,O-chelating coordination mode with Cu—N = 1.9814 (18) Å.

The pyrazolate ring and one Cl atom bridge two Cu^II^ ions. The intermetallic separation Cu1—Cu2 is 3.9067 (4) Å, which is similar to that seen in the structure reported by King *et al.* (2004) (3.962 Å).

The crystal packing is presented in Fig. 2. An O—H···O hydrogen bond connects the complex molecule and propan-2-ol solvent molecule in pair. These units are stacked along the crystallographic *b* axis, forming a column-like structure. The two pyridine molecules interact through an intramolecular π–stacking interaction with a distance of 3.869 (1) Å between the centroids of the pyridine rings in the complex.

### Experimental

Copper(II) chloride dihydrate (0.05 g, 0.29 mmol) was dissolved in DMF (4 ml), and mixed with solution of L (0.078 g, 0.29 mmol) in DMF (3 ml). Then to the reaction mixture pyridine was added within 24 h . Blue block-shaped crystals of the title compound were obtained upon slow diffusion of propan-2-ol vapour into dark-green solution during two weeks (the solution turns blue over time). Analysis calculated for C_30_H_34_Cl_2_Cu_2_N_6_O_4_: C 48.61, H 4.59, N 11.34%; found: C 48.45, H 4.70, N 11.43%.

### Refinement

H atom attached to O atom was located from the difference Fourier map, and refined with *U*_iso_ = 1.5 *U*_eq_(O). The remaining H atoms were positioned geometrically and refined as riding atoms, with C—H = 0.95–1.00 Å and with *U*_iso_ = 1.2(1.5 for methyl)*U*_eq_(C).

### Crystal data

**Table:**

[Cu_2_(C_7_H_6_N_2_O_3_)Cl_2_(C_5_H_5_N)_4_]·C_3_H_8_O	*F*(000) = 1520
*M**_r_* = 740.61	*D*_x_ = 1.498 Mg m^−^^3^
Monoclinic, *P*2_1_/*c*	Mo *K*α radiation, λ = 0.71073 Å
Hall symbol: -P 2ybc	Cell parameters from 14278 reflections
*a* = 16.4130 (4) Å	θ = 1.0–30.0°
*b* = 12.6351 (2) Å	µ = 1.50 mm^−^^1^
*c* = 16.5739 (4) Å	*T* = 100 K
β = 107.2145 (12)°	Block, green-blue
*V* = 3283.12 (12) Å^3^	0.24 × 0.16 × 0.13 mm
*Z* = 4	

### Data collection

**Table:**

Nonius KappaCCD diffractometer	8719 independent reflections
Radiation source: fine-focus sealed tube	6355 reflections with *I* > 2σ(*I*)
horizontally mounted graphite crystal	*R*_int_ = 0.058
Detector resolution: 9 pixels mm^-1^	θ_max_ = 29.0°, θ_min_ = 2.6°
φ and ω scans with κ offset	*h* = −22→22
Absorption correction: multi-scan (*SADABS*; Sheldrick, 1996)	*k* = −17→17
*T*_min_ = 0.718, *T*_max_ = 0.832	*l* = −21→22
45344 measured reflections	

### Refinement

**Table:**

Refinement on *F*^2^	Primary atom site location: structure-invariant direct methods
Least-squares matrix: full	Secondary atom site location: difference Fourier map
*R*[*F*^2^ > 2σ(*F*^2^)] = 0.038	Hydrogen site location: inferred from neighbouring sites
*wR*(*F*^2^) = 0.086	H-atom parameters constrained
*S* = 1.03	*w* = 1/[σ^2^(*F*_o_^2^) + (0.0362*P*)^2^ + 1.5874*P*] where *P* = (*F*_o_^2^ + 2*F*_c_^2^)/3
8719 reflections	(Δ/σ)_max_ = 0.001
402 parameters	Δρ_max_ = 0.59 e Å^−^^3^
0 restraints	Δρ_min_ = −0.47 e Å^−^^3^

### Fractional atomic coordinates and isotropic or equivalent isotropic displacement parameters (Å^2^)

**Table:**

	*x*	*y*	*z*	*U*_iso_*/*U*_eq_	
Cu1	0.152430 (17)	−0.09197 (2)	0.372827 (16)	0.01651 (7)	
Cu2	0.227801 (17)	0.202366 (19)	0.406284 (16)	0.01633 (7)	
Cl1	0.03110 (3)	−0.17696 (4)	0.37302 (3)	0.02075 (12)	
Cl2	0.09566 (3)	0.12326 (4)	0.35994 (3)	0.01988 (12)	
O1	0.25807 (11)	−0.24098 (12)	0.36521 (10)	0.0256 (4)	
O2	0.34674 (10)	0.26096 (12)	0.42092 (9)	0.0216 (3)	
O3	0.47931 (11)	0.21282 (13)	0.42382 (11)	0.0305 (4)	
H3O	0.5189	0.2845	0.3481	0.046*	
O4	0.55635 (13)	0.32508 (17)	0.32569 (13)	0.0460 (5)	
N1	0.26482 (11)	−0.02909 (13)	0.37947 (11)	0.0167 (4)	
N2	0.28640 (12)	0.07195 (13)	0.39256 (11)	0.0165 (4)	
N3	0.12367 (12)	−0.10132 (14)	0.24327 (11)	0.0181 (4)	
N4	0.18472 (12)	−0.08623 (13)	0.50126 (11)	0.0186 (4)	
N5	0.18432 (12)	0.35174 (14)	0.38697 (11)	0.0191 (4)	
N6	0.24312 (12)	0.20815 (14)	0.54257 (11)	0.0207 (4)	
C1	0.33529 (14)	−0.08399 (17)	0.37618 (13)	0.0179 (4)	
C2	0.32611 (15)	−0.19962 (17)	0.36623 (14)	0.0208 (5)	
C3	0.40018 (16)	−0.26389 (19)	0.36067 (16)	0.0293 (6)	
H3A	0.3827	−0.3380	0.3498	0.044*	
H3B	0.4462	−0.2588	0.4140	0.044*	
H3C	0.4204	−0.2372	0.3145	0.044*	
C4	0.40414 (14)	−0.01443 (18)	0.38640 (13)	0.0202 (5)	
C5	0.49426 (15)	−0.0362 (2)	0.38719 (15)	0.0266 (5)	
H5A	0.5251	0.0308	0.3904	0.040*	
H5B	0.4941	−0.0738	0.3354	0.040*	
H5C	0.5225	−0.0800	0.4363	0.040*	
C6	0.36967 (14)	0.08353 (17)	0.39700 (13)	0.0180 (5)	
C7	0.40334 (15)	0.19301 (18)	0.41462 (13)	0.0210 (5)	
C8	0.09276 (14)	−0.19093 (17)	0.20156 (14)	0.0205 (5)	
H8	0.0852	−0.2509	0.2332	0.025*	
C9	0.07149 (15)	−0.19954 (18)	0.11467 (15)	0.0240 (5)	
H9	0.0503	−0.2644	0.0874	0.029*	
C10	0.08148 (16)	−0.1127 (2)	0.06806 (15)	0.0275 (5)	
H10	0.0667	−0.1163	0.0082	0.033*	
C11	0.11359 (16)	−0.02000 (19)	0.11047 (15)	0.0290 (6)	
H11	0.1212	0.0411	0.0801	0.035*	
C12	0.13440 (15)	−0.01797 (18)	0.19762 (15)	0.0236 (5)	
H12	0.1573	0.0454	0.2264	0.028*	
C13	0.13414 (15)	−0.03639 (17)	0.53964 (14)	0.0220 (5)	
H13	0.0846	−0.0015	0.5056	0.026*	
C14	0.15093 (16)	−0.03359 (19)	0.62622 (15)	0.0276 (6)	
H14	0.1138	0.0029	0.6511	0.033*	
C15	0.22260 (18)	−0.0847 (2)	0.67623 (16)	0.0328 (6)	
H15	0.2354	−0.0844	0.7360	0.039*	
C16	0.27560 (17)	−0.1365 (2)	0.63733 (16)	0.0335 (6)	
H16	0.3254	−0.1721	0.6701	0.040*	
C17	0.25461 (16)	−0.13519 (19)	0.55032 (15)	0.0264 (5)	
H17	0.2911	−0.1704	0.5240	0.032*	
C18	0.23103 (16)	0.42893 (18)	0.43517 (14)	0.0247 (5)	
H18	0.2827	0.4103	0.4767	0.030*	
C19	0.20695 (16)	0.53372 (17)	0.42661 (15)	0.0261 (5)	
H19	0.2411	0.5861	0.4621	0.031*	
C20	0.13255 (16)	0.56161 (18)	0.36572 (14)	0.0241 (5)	
H20	0.1146	0.6334	0.3588	0.029*	
C21	0.08464 (14)	0.48361 (17)	0.31503 (14)	0.0202 (5)	
H21	0.0335	0.5009	0.2722	0.024*	
C22	0.11246 (14)	0.37959 (17)	0.32779 (13)	0.0183 (5)	
H22	0.0792	0.3259	0.2931	0.022*	
C23	0.31973 (18)	0.1915 (2)	0.59788 (16)	0.0394 (7)	
H23	0.3664	0.1773	0.5767	0.047*	
C24	0.33449 (19)	0.1939 (3)	0.68440 (16)	0.0445 (8)	
H24	0.3899	0.1810	0.7217	0.053*	
C25	0.26752 (18)	0.2153 (2)	0.71528 (16)	0.0342 (6)	
H25	0.2755	0.2164	0.7744	0.041*	
C26	0.18849 (16)	0.23518 (19)	0.65927 (15)	0.0273 (5)	
H26	0.1414	0.2526	0.6791	0.033*	
C27	0.17882 (15)	0.22930 (17)	0.57336 (14)	0.0214 (5)	
H27	0.1238	0.2409	0.5349	0.026*	
C28	0.6574 (2)	0.4623 (3)	0.3462 (2)	0.0615 (10)	
H28A	0.7052	0.4127	0.3547	0.092*	
H28B	0.6775	0.5276	0.3776	0.092*	
H28C	0.6342	0.4785	0.2859	0.092*	
C29	0.58876 (19)	0.4128 (2)	0.37756 (18)	0.0424 (7)	
H29	0.6153	0.3871	0.4366	0.051*	
C30	0.5193 (3)	0.4877 (3)	0.3776 (3)	0.0765 (12)	
H30A	0.4981	0.5210	0.3219	0.115*	
H30B	0.5411	0.5422	0.4206	0.115*	
H30C	0.4727	0.4492	0.3903	0.115*	

### Atomic displacement parameters (Å^2^)

**Table:**

	*U*^11^	*U*^22^	*U*^33^	*U*^12^	*U*^13^	*U*^23^
Cu1	0.01760 (15)	0.01758 (14)	0.01463 (14)	−0.00056 (10)	0.00519 (11)	−0.00005 (10)
Cu2	0.01967 (15)	0.01512 (13)	0.01380 (14)	0.00081 (10)	0.00435 (11)	−0.00014 (10)
Cl1	0.0200 (3)	0.0225 (3)	0.0202 (3)	−0.0027 (2)	0.0066 (2)	0.0001 (2)
Cl2	0.0195 (3)	0.0164 (2)	0.0236 (3)	0.0010 (2)	0.0061 (2)	0.0000 (2)
O1	0.0276 (10)	0.0197 (8)	0.0289 (9)	0.0014 (7)	0.0077 (8)	−0.0007 (7)
O2	0.0233 (9)	0.0209 (8)	0.0198 (8)	−0.0023 (7)	0.0049 (7)	−0.0015 (6)
O3	0.0215 (9)	0.0342 (9)	0.0352 (10)	−0.0071 (7)	0.0076 (8)	−0.0003 (8)
O4	0.0422 (12)	0.0577 (13)	0.0413 (12)	−0.0113 (10)	0.0171 (10)	−0.0037 (10)
N1	0.0183 (10)	0.0164 (9)	0.0145 (9)	0.0024 (7)	0.0036 (8)	0.0000 (7)
N2	0.0176 (10)	0.0176 (9)	0.0139 (9)	−0.0010 (7)	0.0039 (7)	0.0000 (7)
N3	0.0175 (10)	0.0202 (9)	0.0170 (9)	0.0015 (7)	0.0055 (8)	0.0003 (7)
N4	0.0209 (10)	0.0178 (9)	0.0172 (9)	−0.0029 (7)	0.0057 (8)	0.0006 (7)
N5	0.0263 (11)	0.0173 (9)	0.0134 (9)	0.0013 (8)	0.0053 (8)	0.0004 (7)
N6	0.0218 (10)	0.0239 (10)	0.0162 (9)	0.0068 (8)	0.0052 (8)	0.0009 (7)
C1	0.0181 (11)	0.0231 (11)	0.0119 (10)	0.0032 (9)	0.0037 (9)	−0.0011 (8)
C2	0.0244 (13)	0.0216 (11)	0.0148 (11)	0.0051 (10)	0.0034 (9)	−0.0008 (9)
C3	0.0271 (14)	0.0269 (12)	0.0322 (14)	0.0080 (10)	0.0061 (11)	−0.0076 (10)
C4	0.0195 (12)	0.0276 (12)	0.0132 (11)	0.0024 (9)	0.0044 (9)	0.0003 (9)
C5	0.0177 (12)	0.0361 (14)	0.0256 (13)	0.0032 (10)	0.0061 (10)	−0.0036 (10)
C6	0.0196 (12)	0.0230 (11)	0.0112 (10)	0.0000 (9)	0.0042 (9)	0.0001 (8)
C7	0.0218 (13)	0.0269 (12)	0.0128 (11)	−0.0027 (10)	0.0029 (9)	0.0034 (9)
C8	0.0229 (12)	0.0190 (11)	0.0204 (11)	0.0013 (9)	0.0077 (10)	0.0005 (9)
C9	0.0236 (13)	0.0240 (12)	0.0252 (12)	−0.0005 (10)	0.0085 (10)	−0.0066 (10)
C10	0.0279 (14)	0.0381 (14)	0.0177 (12)	−0.0016 (11)	0.0085 (10)	−0.0013 (10)
C11	0.0365 (15)	0.0288 (13)	0.0214 (12)	−0.0064 (11)	0.0083 (11)	0.0038 (10)
C12	0.0256 (13)	0.0231 (12)	0.0232 (12)	−0.0044 (10)	0.0087 (10)	0.0002 (9)
C13	0.0265 (13)	0.0188 (11)	0.0218 (12)	0.0001 (9)	0.0088 (10)	0.0011 (9)
C14	0.0382 (16)	0.0264 (12)	0.0223 (12)	−0.0020 (11)	0.0151 (12)	−0.0036 (10)
C15	0.0403 (16)	0.0418 (15)	0.0165 (12)	−0.0052 (12)	0.0084 (11)	−0.0013 (11)
C16	0.0316 (15)	0.0447 (16)	0.0214 (13)	0.0066 (12)	0.0035 (11)	0.0069 (11)
C17	0.0242 (13)	0.0336 (13)	0.0209 (12)	0.0032 (10)	0.0060 (10)	−0.0003 (10)
C18	0.0301 (14)	0.0225 (12)	0.0172 (11)	−0.0003 (10)	0.0002 (10)	−0.0012 (9)
C19	0.0363 (15)	0.0171 (11)	0.0219 (12)	−0.0024 (10)	0.0040 (11)	−0.0043 (9)
C20	0.0356 (14)	0.0169 (11)	0.0236 (12)	0.0017 (10)	0.0146 (11)	0.0046 (9)
C21	0.0192 (12)	0.0238 (11)	0.0189 (11)	0.0024 (9)	0.0076 (9)	0.0055 (9)
C22	0.0194 (12)	0.0206 (11)	0.0157 (11)	−0.0024 (9)	0.0064 (9)	−0.0002 (8)
C23	0.0300 (15)	0.072 (2)	0.0174 (13)	0.0209 (14)	0.0083 (11)	0.0048 (13)
C24	0.0322 (16)	0.080 (2)	0.0190 (13)	0.0164 (15)	0.0046 (12)	0.0038 (14)
C25	0.0401 (16)	0.0475 (16)	0.0159 (12)	−0.0050 (13)	0.0095 (12)	−0.0070 (11)
C26	0.0268 (14)	0.0343 (13)	0.0254 (13)	−0.0073 (11)	0.0147 (11)	−0.0083 (10)
C27	0.0211 (12)	0.0210 (11)	0.0220 (12)	−0.0021 (9)	0.0064 (10)	−0.0022 (9)
C28	0.063 (2)	0.090 (3)	0.0310 (17)	−0.040 (2)	0.0120 (16)	−0.0058 (17)
C29	0.0398 (17)	0.0536 (18)	0.0330 (16)	−0.0096 (14)	0.0097 (13)	−0.0041 (13)
C30	0.082 (3)	0.056 (2)	0.095 (3)	0.008 (2)	0.030 (2)	−0.010 (2)

### Geometric parameters (Å, °)

**Table:**

Cu1—N1	1.9814 (18)	C9—H9	0.9500
Cu1—N3	2.0609 (18)	C10—C11	1.387 (3)
Cu1—N4	2.0371 (18)	C10—H10	0.9500
Cu1—O1	2.5878 (17)	C11—C12	1.383 (3)
Cu1—Cl1	2.2634 (6)	C11—H11	0.9500
Cu1—Cl2	2.8621 (6)	C12—H12	0.9500
Cu2—N2	1.9549 (18)	C13—C14	1.379 (3)
Cu2—N5	2.0097 (18)	C13—H13	0.9500
Cu2—N6	2.1987 (18)	C14—C15	1.382 (4)
Cu2—O2	2.0340 (16)	C14—H14	0.9500
Cu2—Cl2	2.3036 (6)	C15—C16	1.390 (4)
O1—C2	1.229 (3)	C15—H15	0.9500
O2—C7	1.292 (3)	C16—C17	1.380 (3)
O3—C7	1.236 (3)	C16—H16	0.9500
O4—C29	1.407 (3)	C17—H17	0.9500
O4—H3O	0.9550	C18—C19	1.377 (3)
N1—N2	1.325 (2)	C18—H18	0.9500
N1—C1	1.364 (3)	C19—C20	1.380 (3)
N2—C6	1.355 (3)	C19—H19	0.9500
N3—C12	1.338 (3)	C20—C21	1.380 (3)
N3—C8	1.345 (3)	C20—H20	0.9500
N4—C13	1.343 (3)	C21—C22	1.387 (3)
N4—C17	1.344 (3)	C21—H21	0.9500
N5—C22	1.339 (3)	C22—H22	0.9500
N5—C18	1.347 (3)	C23—C24	1.382 (4)
N6—C27	1.328 (3)	C23—H23	0.9500
N6—C23	1.335 (3)	C24—C25	1.370 (4)
C1—C4	1.402 (3)	C24—H24	0.9500
C1—C2	1.473 (3)	C25—C26	1.376 (4)
C2—C3	1.487 (3)	C25—H25	0.9500
C3—H3A	0.9800	C26—C27	1.387 (3)
C3—H3B	0.9800	C26—H26	0.9500
C3—H3C	0.9800	C27—H27	0.9500
C4—C6	1.393 (3)	C28—C29	1.509 (4)
C4—C5	1.501 (3)	C28—H28A	0.9800
C5—H5A	0.9800	C28—H28B	0.9800
C5—H5B	0.9800	C28—H28C	0.9800
C5—H5C	0.9800	C29—C30	1.482 (5)
C6—C7	1.486 (3)	C29—H29	1.0000
C8—C9	1.382 (3)	C30—H30A	0.9800
C8—H8	0.9500	C30—H30B	0.9800
C9—C10	1.379 (3)	C30—H30C	0.9800
			
N1—Cu1—N4	88.72 (7)	C10—C9—C8	119.1 (2)
N1—Cu1—N3	90.19 (7)	C10—C9—H9	120.5
N4—Cu1—N3	177.86 (7)	C8—C9—H9	120.5
N1—Cu1—Cl1	174.40 (5)	C9—C10—C11	118.6 (2)
N4—Cu1—Cl1	88.41 (5)	C9—C10—H10	120.7
N3—Cu1—Cl1	92.52 (5)	C11—C10—H10	120.7
N1—Cu1—O1	70.62 (7)	C12—C11—C10	119.0 (2)
N3—Cu1—O1	81.82 (6)	C12—C11—H11	120.5
N4—Cu1—O1	96.09 (6)	C10—C11—H11	120.5
Cl1—Cu1—O1	104.91 (4)	N3—C12—C11	122.9 (2)
Cl2—Cu1—O1	153.60 (4)	N3—C12—H12	118.6
N1—Cu1—Cl2	84.28 (5)	C11—C12—H12	118.6
N3—Cu1—Cl2	90.44 (5)	N4—C13—C14	122.9 (2)
N4—Cu1—Cl2	91.29 (5)	N4—C13—H13	118.5
Cl1—Cu1—Cl2	100.59 (2)	C14—C13—H13	118.5
N2—Cu2—N5	159.53 (7)	C13—C14—C15	119.0 (2)
N2—Cu2—O2	80.38 (7)	C13—C14—H14	120.5
N5—Cu2—O2	87.72 (7)	C15—C14—H14	120.5
N2—Cu2—N6	103.71 (7)	C14—C15—C16	118.7 (2)
N5—Cu2—N6	93.42 (7)	C14—C15—H15	120.7
O2—Cu2—N6	92.95 (7)	C16—C15—H15	120.7
N2—Cu2—Cl2	92.44 (5)	C17—C16—C15	118.8 (2)
N5—Cu2—Cl2	95.71 (6)	C17—C16—H16	120.6
O2—Cu2—Cl2	166.73 (5)	C15—C16—H16	120.6
N6—Cu2—Cl2	99.62 (5)	N4—C17—C16	122.8 (2)
C7—O2—Cu2	115.87 (14)	N4—C17—H17	118.6
C29—O4—H3O	110.8	C16—C17—H17	118.6
N2—N1—C1	107.93 (18)	N5—C18—C19	122.6 (2)
N2—N1—Cu1	126.48 (14)	N5—C18—H18	118.7
C1—N1—Cu1	125.49 (14)	C19—C18—H18	118.7
N1—N2—C6	109.22 (17)	C18—C19—C20	119.1 (2)
N1—N2—Cu2	135.57 (15)	C18—C19—H19	120.5
C6—N2—Cu2	115.20 (14)	C20—C19—H19	120.5
C12—N3—C8	117.68 (19)	C21—C20—C19	119.0 (2)
C12—N3—Cu1	121.06 (15)	C21—C20—H20	120.5
C8—N3—Cu1	121.26 (15)	C19—C20—H20	120.5
C13—N4—C17	117.8 (2)	C20—C21—C22	118.8 (2)
C13—N4—Cu1	120.37 (15)	C20—C21—H21	120.6
C17—N4—Cu1	121.81 (15)	C22—C21—H21	120.6
C22—N5—C18	117.88 (19)	N5—C22—C21	122.7 (2)
C22—N5—Cu2	123.91 (15)	N5—C22—H22	118.7
C18—N5—Cu2	118.20 (15)	C21—C22—H22	118.7
C27—N6—C23	117.5 (2)	N6—C23—C24	123.3 (2)
C27—N6—Cu2	122.62 (15)	N6—C23—H23	118.3
C23—N6—Cu2	119.87 (16)	C24—C23—H23	118.3
N1—C1—C4	109.83 (19)	C25—C24—C23	118.6 (3)
N1—C1—C2	116.7 (2)	C25—C24—H24	120.7
C4—C1—C2	133.4 (2)	C23—C24—H24	120.7
O1—C2—C1	119.1 (2)	C24—C25—C26	119.0 (2)
O1—C2—C3	121.5 (2)	C24—C25—H25	120.5
C1—C2—C3	119.3 (2)	C26—C25—H25	120.5
C2—C3—H3A	109.5	C25—C26—C27	118.8 (2)
C2—C3—H3B	109.5	C25—C26—H26	120.6
H3A—C3—H3B	109.5	C27—C26—H26	120.6
C2—C3—H3C	109.5	N6—C27—C26	122.9 (2)
H3A—C3—H3C	109.5	N6—C27—H27	118.6
H3B—C3—H3C	109.5	C26—C27—H27	118.6
C6—C4—C1	103.25 (19)	C29—C28—H28A	109.5
C6—C4—C5	126.7 (2)	C29—C28—H28B	109.5
C1—C4—C5	130.0 (2)	H28A—C28—H28B	109.5
C4—C5—H5A	109.5	C29—C28—H28C	109.5
C4—C5—H5B	109.5	H28A—C28—H28C	109.5
H5A—C5—H5B	109.5	H28B—C28—H28C	109.5
C4—C5—H5C	109.5	O4—C29—C30	110.6 (3)
H5A—C5—H5C	109.5	O4—C29—C28	107.5 (2)
H5B—C5—H5C	109.5	C30—C29—C28	112.9 (3)
N2—C6—C4	109.76 (19)	O4—C29—H29	108.6
N2—C6—C7	114.94 (19)	C30—C29—H29	108.6
C4—C6—C7	135.3 (2)	C28—C29—H29	108.6
O3—C7—O2	125.4 (2)	C29—C30—H30A	109.5
O3—C7—C6	121.0 (2)	C29—C30—H30B	109.5
O2—C7—C6	113.6 (2)	H30A—C30—H30B	109.5
N3—C8—C9	122.8 (2)	C29—C30—H30C	109.5
N3—C8—H8	118.6	H30A—C30—H30C	109.5
C9—C8—H8	118.6	H30B—C30—H30C	109.5
			
N2—Cu2—O2—C7	0.53 (15)	C4—C1—C2—C3	−3.8 (4)
N5—Cu2—O2—C7	−162.76 (15)	N1—C1—C4—C6	0.8 (2)
N6—Cu2—O2—C7	103.94 (15)	C2—C1—C4—C6	−176.6 (2)
Cl2—Cu2—O2—C7	−57.4 (3)	N1—C1—C4—C5	−179.8 (2)
N4—Cu1—N1—N2	−78.06 (17)	C2—C1—C4—C5	2.8 (4)
N3—Cu1—N1—N2	103.78 (17)	N1—N2—C6—C4	−0.1 (2)
N4—Cu1—N1—C1	97.87 (17)	Cu2—N2—C6—C4	179.96 (14)
N3—Cu1—N1—C1	−80.29 (17)	N1—N2—C6—C7	−177.94 (17)
C1—N1—N2—C6	0.6 (2)	Cu2—N2—C6—C7	2.1 (2)
Cu1—N1—N2—C6	177.10 (14)	C1—C4—C6—N2	−0.5 (2)
C1—N1—N2—Cu2	−179.44 (15)	C5—C4—C6—N2	−179.9 (2)
Cu1—N1—N2—Cu2	−2.9 (3)	C1—C4—C6—C7	176.8 (2)
N5—Cu2—N2—N1	−126.1 (2)	C5—C4—C6—C7	−2.6 (4)
O2—Cu2—N2—N1	178.6 (2)	Cu2—O2—C7—O3	−177.94 (17)
N6—Cu2—N2—N1	87.9 (2)	Cu2—O2—C7—C6	0.4 (2)
Cl2—Cu2—N2—N1	−12.64 (19)	N2—C6—C7—O3	176.8 (2)
N5—Cu2—N2—C6	53.8 (3)	C4—C6—C7—O3	−0.3 (4)
O2—Cu2—N2—C6	−1.45 (14)	N2—C6—C7—O2	−1.6 (3)
N6—Cu2—N2—C6	−92.15 (15)	C4—C6—C7—O2	−178.8 (2)
Cl2—Cu2—N2—C6	167.33 (14)	C12—N3—C8—C9	−0.5 (3)
N1—Cu1—N3—C12	−49.00 (18)	Cu1—N3—C8—C9	178.93 (17)
Cl1—Cu1—N3—C12	135.90 (17)	N3—C8—C9—C10	−0.6 (4)
N1—Cu1—N3—C8	131.61 (17)	C8—C9—C10—C11	0.8 (4)
Cl1—Cu1—N3—C8	−43.49 (17)	C9—C10—C11—C12	0.0 (4)
N1—Cu1—N4—C13	124.85 (17)	C8—N3—C12—C11	1.3 (3)
Cl1—Cu1—N4—C13	−59.96 (16)	Cu1—N3—C12—C11	−178.08 (18)
N1—Cu1—N4—C17	−57.93 (18)	C10—C11—C12—N3	−1.1 (4)
Cl1—Cu1—N4—C17	117.26 (17)	C17—N4—C13—C14	0.0 (3)
N2—Cu2—N5—C22	84.2 (3)	Cu1—N4—C13—C14	177.31 (17)
O2—Cu2—N5—C22	138.41 (18)	N4—C13—C14—C15	−0.4 (4)
N6—Cu2—N5—C22	−128.77 (18)	C13—C14—C15—C16	0.5 (4)
Cl2—Cu2—N5—C22	−28.74 (17)	C14—C15—C16—C17	−0.1 (4)
N2—Cu2—N5—C18	−94.3 (3)	C13—N4—C17—C16	0.4 (3)
O2—Cu2—N5—C18	−40.15 (17)	Cu1—N4—C17—C16	−176.94 (19)
N6—Cu2—N5—C18	52.67 (18)	C15—C16—C17—N4	−0.3 (4)
Cl2—Cu2—N5—C18	152.70 (16)	C22—N5—C18—C19	1.1 (3)
N2—Cu2—N6—C27	−133.26 (17)	Cu2—N5—C18—C19	179.71 (19)
N5—Cu2—N6—C27	58.05 (18)	N5—C18—C19—C20	−0.7 (4)
O2—Cu2—N6—C27	145.94 (17)	C18—C19—C20—C21	−0.2 (4)
Cl2—Cu2—N6—C27	−38.34 (17)	C19—C20—C21—C22	0.8 (3)
N2—Cu2—N6—C23	47.7 (2)	C18—N5—C22—C21	−0.4 (3)
N5—Cu2—N6—C23	−121.0 (2)	Cu2—N5—C22—C21	−179.01 (16)
O2—Cu2—N6—C23	−33.1 (2)	C20—C21—C22—N5	−0.5 (3)
Cl2—Cu2—N6—C23	142.7 (2)	C27—N6—C23—C24	0.9 (4)
N2—N1—C1—C4	−0.9 (2)	Cu2—N6—C23—C24	179.9 (2)
Cu1—N1—C1—C4	−177.46 (14)	N6—C23—C24—C25	−0.6 (5)
N2—N1—C1—C2	177.02 (17)	C23—C24—C25—C26	−1.0 (4)
Cu1—N1—C1—C2	0.5 (3)	C24—C25—C26—C27	2.1 (4)
N1—C1—C2—O1	−3.2 (3)	C23—N6—C27—C26	0.4 (3)
C4—C1—C2—O1	174.1 (2)	Cu2—N6—C27—C26	−178.65 (17)
N1—C1—C2—C3	178.91 (19)	C25—C26—C27—N6	−1.9 (4)

### Hydrogen-bond geometry (Å, °)

**Table:**

*D*—H···*A*	*D*—H	H···*A*	*D*···*A*	*D*—H···*A*
O4—H3O···O3	0.95	1.82	2.734 (3)	160
